# Liquid–liquid phase inclusion bodies in acute and persistent parainfluenaza virus type 5 infections

**DOI:** 10.1099/jgv.0.002021

**Published:** 2024-09-12

**Authors:** E. B. Wignall-Fleming, T. S. Carlos, R. E. Randall

**Affiliations:** 1 School of Biology, Centre for Biomolecular Sciences, BMS Building, North Haugh, University of St. Andrews, St. Andrews, Fife, KY16 9ST, UK

**Keywords:** inclusion bodies, liquid–liquid organelles, paramyxoviruses, parainfluenza virus type 5, persistence

## Abstract

Cytoplasmic inclusion bodies (IBs) are a common feature of single-stranded, non-segmented, negative-strand RNA virus (Mononegavirales) infections and are thought to be regions of active virus transcription and replication. Here we followed the dynamics of IB formation and maintenance in cells infected with persistent and lytic/acute variants of the paramyxovirus, parainfluenza virus type 5 (PIV5). We show that there is a rapid increase in the number of small inclusions bodies up until approximately 12 h post-infection. Thereafter the number of inclusion bodies decreases but they increase in size, presumably due to the fusion of these liquid organelles that can be disrupted by osmotically shocking cells. No obvious differences were observed at these times between inclusion body formation in cells infected with lytic/acute and persistent viruses. IBs are also readily detected in cells persistently infected with PIV5, including in cells in which there is little or no ongoing virus transcription or replication. *In situ* hybridization shows that genomic RNA is primarily located in IBs, whilst viral mRNA is more diffusely distributed throughout the cytoplasm. Some, but not all, IBs show incorporation of 5-ethynyl-uridine (5EU), which is integrated into newly synthesized RNA, at early times post-infection. These results strongly suggest that, although genomic RNA is present in all IBs, IBs are not continuously active sites of virus transcription and replication. Disruption of IBs by osmotically shocking persistently infected cells does not increase virus protein synthesis, suggesting that in persistently infected cells most of the virus genomes are in a repressed state. The role of IBs in PIV5 replication and the establishment and maintenance of persistence is discussed.

## Data Summary

No data presented here are required for submission to open data according to Microbiology Society Guidelines.

## Introduction

Paramyxoviruses are the causative agent of a number of clinically important diseases, including childhood maladies such as mumps and measles, as well as other respiratory illnesses. Paramyxoviruses have pandemic potential and are capable of crossing the species barrier. For example, Nipah and Hendra viruses can jump the species barrier into the human population and exhibit high mortality rates [1,2]. Paramyxoviruses have similar replication and transcription mechanisms to other single-stranded, negative-sense RNA (nsRNA) viruses (Mononegavirales) with a comparable but not identical cohort of genes [3,5]. For paramyxoviruses, the RNA genome is approximately 15–19 kb in size and is encapsidated by the viral nucleoprotein (NP) forming the nucleocapsid complex which provides the template for viral RNA synthesis [5]. The nucleocapsid also shields the viral RNA from recognition by host–pathogen recognition receptors (PRRs) and induction of cellular innate immune responses. The genome has non-coding leader (Le) and trailer (Tr) regions at its 3′ and 5′ ends, respectively, that contain the promoters which are recognized by the viral RNA-dependent RNA polymerase (RdRp), composed of the viral proteins large (L) and phosphoprotein (P), resulting in the initiation of either transcription or replication of the virus genome [6,8]. Upon cell entry, the virus commences transcription making individual mRNAs through a stop–start mechanism which is controlled by *cis*-acting elements positioned at the start and end of each gene [9]. As virus transcription proceeds the virus polymerase may disengage the template resulting in a transcription gradient with genes closest to the Le promoter at the 3′ end of the genome generating the most abundant mRNAs and genes at the 5′ end producing the least abundant mRNAs [10,14]. Following primary transcription, the RdRp can then switch to viral replication; the switch from transcription to replication is widely accepted to be controlled by the abundance of viral protein NP (NP^0^) which is kept soluble through its interactions with viral proteins P and/or V [7,8, 15,18]. During replication, the RdRp ignores the *cis*-acting elements and produces a full-length positive-sense copy of the genome, known as the antigenome, which acts as a template for the synthesis of new virus genomes. During the synthesis of genomic and antigenomic RNA, the nascent strand is concurrently encapsidated by NP^0^ [19,20].

A characteristic of Mononegavirales replication is the formation of viral inclusion bodies (IBs) in the cytoplasm of infected cells. These are believed to facilitate virus replication by concentrating the proteins required for viral RNA synthesis, whilst additionally shielding the nascent viral RNA from triggering cellular innate immune responses. Studies using rabies virus (RABV), vesicular stomatitis virus (VSV), respiratory syncytial virus (RSV), Ebola virus (EBOV), Nipah virus (NiV), measles virus (MeV), mumps virus (MuV), human metapneumovirus (HMPV), and parainfluenza virus 3 and 5 (PIV3 and PIV5) have shown IBs to have the properties of membrane-less liquid organelles which are formed by liquid–liquid phase separation (LLPS) [21,34], similar to other eukaryotic cellular structures such as stress granules and P-bodies [35,38]. The minimum requirement for the generation of viral IBs generated during most Mononegavirales infection, including PIV5, are the viral proteins NP and P [16]. Using *in situ* hybridization, we have shown that for PIV5, as have others for PIV3, RABV and RSV, viral genomes are located within IBs [25,29, 33, 34, 39,41]. Additionally, studies of a number of Mononegavirales, including EBOV, RSV and RABV, have shown that *de novo* viral RNA synthesis occurs primarily in IBs [22,29, 31, 40]. From such studies it has been suggested that virus replication and production of virus nucleocapsids occurs within IBs. Newly synthesized nucleocapsids are subsequently thought to be exported from the IBs to other sites in the cytoplasm, via the cellular cytoskeleton, resulting in the formation of new IBs or, alternatively, to the cell membrane for assembly into virus particles [21,25, 39].

PIV5 (species *Mammalian rubulavirus 5*) is a prototypic member of the rubulavirinae subfamily in the family *Paramyxoviridae* that belongs to the order *Mononegavirales*. It is unusual in that it readily crosses species boundaries and has been isolated from a wide variety of mammals, including dogs, cattle, pigs, monkeys and humans. As well as causing acute/lytic infections *in vivo*, there is evidence that it can also cause persistent infections [42,43]. Some strains of PIV5 readily establish persistent infections in cell culture. Persistently infected cells exhibit little or no cytopathic effect (CPE) and can readily be passaged. In persistently infected cells the virus fluxes between active and repressed states. We have shown that the phosphorylation state of the P protein determines whether a particular strain of PIV5 establishes a persistent or lytic/acute infection [42]. We have also shown that PIV5 IBs can be readily detected in persistently infected cells in which there is little or no active ongoing virus protein synthesis, suggesting that IBs may be sites of virus persistence [43]. Here we have extended our observation on PIV5 IBs and discuss their relevance in both acutely and persistently infected cells.

## Methods

### Cells and viruses

All cells were cultured at 37 °C in a 5% CO_2_ atmosphere. A549 cells (ATCC) were maintained in Dulbecco’s modified Eagle’s Medium (DMEM) supplemented with 10% FCS and penicillin and streptomycin (pen/strep). The viruses PIV5 strain W3A (PIV5.S157), PIV5 strain W3A (PIV5.F157) and PIV5 (CPI) were grown as appropriate. Briefly, to make the virus stock vero cells were infected at an m.o.i. of 0.01 p.f.u. per cell and incubated at 37 °C for 3 days. The supernatant was then collected and clarified by centrifugation. The virus stock was then titrated on vero cells.

### Immunofluorescence

Cell monolayers were grown on glass coverslips and were or were not infected with PIV5.S157 and PIV5.F157 at an m.o.i. 10 or 0.1 p.f.u. per cell where appropriate in DMEM supplemented with 2% FCS and pen/strep. The samples were placed on a rocker at 37 °C for 1 h. The absorption medium was then removed and replaced with DMEM supplemented with 10% FCS and pen/strep. The cells were incubated for a specified amount of time before being fixed and permeabilized with 5% formaldehyde in PBS and 0.5% IGEPAL, 10% sucrose in PBS. The cells were then washed and blocked with PBS supplemented with 5% FCS, then incubated with primary antibodies for 1 h at room temperature. After which, the cells were washed as before and incubated with an appropriate secondary antibody and DAPI for 1 h at room temperature. The coverslips were washed with PBS and mounted onto slides using citifluor AF-1 mounting solution (Citifluor). The cells were visualized using an Evos M5000 microscope (Thermo Fisher). Stacked images were taken from three independent areas of the coverslip and analysed using a particle analyser (Fuji).

### RNAscope and *in situ* hybridization

A549 cells were grown in monolayers on glass coverslips. The cells were infected at an m.o.i. of 10 p.f.u. per cell in DMEM supplemented with 2% FCS and pen/strep and placed on a rocker at 37 °C for 1 h. The absorption medium was removed and replaced with DMEM supplemented with 10% FCS and pen/strep. After an appropriate incubation period the cells were fixed using 10% formaldehyde in PBS. The cells were first permeabilized as before for 15 min at room temperature and incubated with anti-NP antibody at 4 °C overnight. The cells were then subjected to RNAscope Multiplex Fluorescent Assay V2 (ACD bio) following the manufacturer’s protocol for adherent cells. A brief description of the protocol is provided. First, cellular peroxidases were removed from the cells by treatment with hydrogen peroxidase for 10 min at room temperature. The cells were then treated with protease III diluted with PBS for 10 min at room temperature. All subsequent incubations were conducted at 40 °C in a humidified chamber provided by ACD known as the Hybez oven. Probes against target RNAs, viral genome and NP mRNA/antigenome were added to the cells and incubated for 2 h. The cells were washed twice with RNA wash buffer, then incubated consecutively with amplification reagents Amp1 and Amp2 for 30 min and Amp 3 for 15 min. The cells were washed twice with RNA wash buffer after each incubation.

To identify individual target RNAs, the HRP molecule HRP-C1, which exclusively recognizes PIV5 genome, was added and incubated for 15 min. A fluorescent dye, opal dye 620 (Akoya Biosciences), was then added and incubated for 30 min. The cells were then washed twice with RNA wash buffer and incubated with an HRP blocker for 15 min.

The cells were then washed with PBS supplemented with 5% FCS. Following this the cells were incubated with anti-mouse FITC secondary antibody and DAPI for 1 h at room temperature. The cells were washed with PBS and mounted onto slides using prolong gold anti-fade (Thermo Fisher Scientific) and visualized using the Evos M5000 (Thermo Fisher Scientific) where stacked images of 0.5 µm slices were obtained and analysed using a particle analyser (Fiji).

The method used for *in situ* hybridization has been described in detail elsewhere [34].

### *De novo* RNA synthesis

A549 cells were grown on coverslips and were infected with PIV5 at an m.o.i. of 10 p.f.u. per cell in DMEM supplemented with 2% FCS and pen/strep and placed in a rocker for 1 h. The medium was then changed to DMEM supplemented with 10% FCS and pen/strep. At either 12 or 24 h post-infection (h.p.i.) cells were then incubated with media containing 1 mM 5-ethynyl-uridine (5EU), using the Click-IT RNA imaging kit (Invitrogen) following the manufacturer’s protocol, for 1 h and then fixed with 10% formaldehyde in PBS. Following this, immunofluorescence was conducted as previously described.

### Quantitative PCR

Monolayers of A549 cells were grown in 60 mm^2^ dishes and were or were not infected with PIV5 WT at an m.o.i. of 10 p.f.u. per cell. The cells were placed on a rocker for 1 h. Virus absorption was conducted at 4 °C to allow the virus to bind but not enter the cells. The infection medium was removed and replaced with pre-warmed DMEM supplemented with 10% FCS and pen/strep allowing the viruses to enter the cells. At the appropriated time after infection the cells were washed with PBS and trizol was added to the cells. Total RNA was extracted using Direct-zol MiniPrep Plus (Zymo) as per the manufacturer’s instructions and quantified by nanodrop. In total, 100 µg of RNA was reverse transcribed using the GoScript reverse transcription (RT) system (Promega). RT was conducted using a single positive-sense primer to recognize exclusively negative-sense genomic RNA in the HN-L intergenic region into the L gene; this region produces the least abundant mRNAs, ensuring that any contaminating L mRNAs would be negligible. Additionally, primers to recognize the housekeeping gene were used to normalize samples to actin. The cDNA was subjected to real-time quantitative PCR (qPCR) using SYBR-Green Master mix (Bio-Rad). After activation of the polymerase for 5 min at 95 °C, the cDNA underwent denaturation for 15 s at 95 °C and annealing/extension for 1 min at 65 °C for 40 cycles. Real-time qPCR was analysed by a Stratagene Mx3005p thermocycler. Cycle threshold (Ct) values of the uninfected mock cells and the PIV5-infected cells were normalized to the housekeeping gene actin to ascertain the ΔCt values. The ΔCt values of the infected cells at different time points were then compared to those of the uninfected mock cells to determine the ΔΔCt fold difference.

### Osmotic shock

A549 cells were seeded and grown on coverslips and infected with PIV5.F157 or PIV5.S157 at an m.o.i. 10 p.f.u. ml^–1^. At 24 h.p.i., or 96 h.p.i. in the case of peresistently infected A549 cells, the cells were incubated with diluted DMEM and water at a ratio of 1 : 5 for 10 min. Where specified the cells were allowed to recover by an additional incubation with DMEM for the stated time. The cells were then fixed, permeabilized and subjected to immunofluorescence as described above.

## Results

### Characterization of IBs during PIV5 infection

**Fig. 1.**
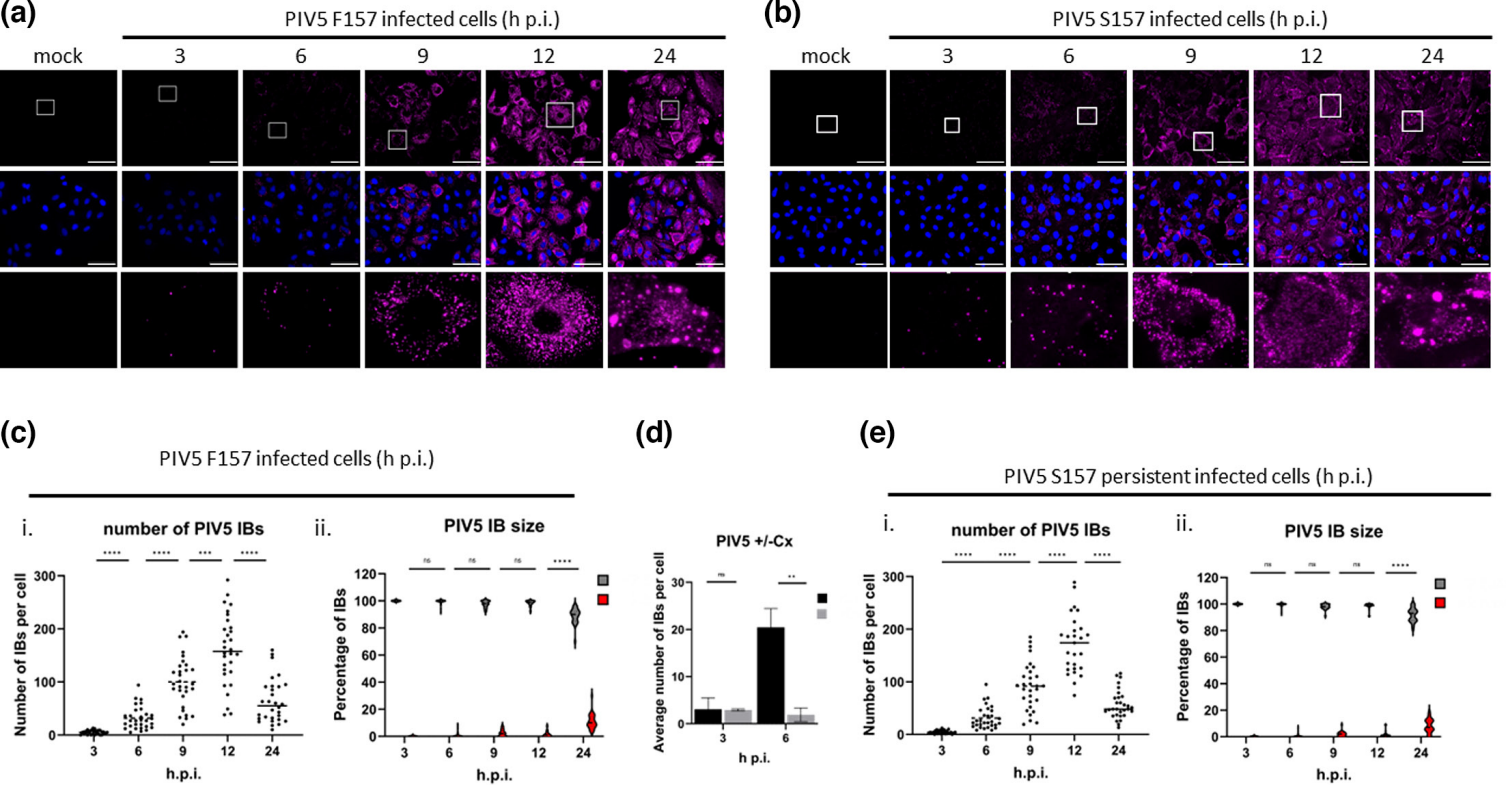
The dynamics of IBs formed during lytic/acute and persistent PIV5 infections over time. (**a, b**) A549 cells seeded on coverslips were infected with PIV5.F157 (lytic/acute) or PIV5.S157 (persistent) at an m.o.i. of 10 p.f.u. per cell. At the indicated time points post-infection the cells were fixed, permeabilized and incubated with an anti-NP mAb. The cells were imaged using the Evos M5000. Bar, 75 µm. (**c, e**) The IBs formed during PIV5-F157 or PIV5-S157 infection were quantified either by number per cell (**i**) or by size (ii) using an ImageJ particle analyser. (**d**) A549 cells grown on coverslips infected with PIV5-S157 at an m.o.i. of 10 p.f.u. per cell in the presence or absence of cycloheximide. The coverslips were fixed, permeabilized and incubated with anti-NP antibody after which the number of IBs was determined as previously described. Images are representative of *n*=3.

PIV5 strains can have either persistent or lytic/acute phenotypes depending upon whether critical amino acids in P can be phosphorylated or not. For example, the W3 strain of PIV5 has a persistent phenotype, but substitution of the serine at position 157 (S157) to a phenylalanine (F157) switches it to a lytic/acute phenotype. To follow the kinetics of formation of PIV5 IBs and investigate whether there were significant differences in IB formation in lytic/acute and persistent infections, A549 cells were infected at a high m.o.i. with either PIV5.S157 or PIV5.F157. At various times post-infection the cells were fixed and immunostained with an anti-NP antibody to visualize IBs (Fig. 1a, b). In PIV5.F157- and PIV5.S157-infected cells IBs were counted and classified according to their size as being either small (≤2.5 µm) or large IBs (>2.5 µm) (Fig. 1c, e). Initially, at 3 h.p.i. only a few small IBs (on average fewer than six per cell) were detectable in both the PIV5.S157- and PIV5.F157-infected cells. Thereafter, the number of IBs increased significantly (Fig. 1). To determine whether the increased number of IBs was dependent upon virus protein synthesis, and hence virus transcription, PIV5-infected A549 cells were treated with cycloheximide (CHX: a protein synthesis inhibitor) immediately after virus adsorption At 6 h.p.i., CHX-treated cells showed no increase in IB number per cell, compared to non-treated PIV5-infected cells in which IBs increased to ~20 per cell, indicating that the increase in IB number is dependent upon virus protein synthesis (Fig. 1d). In the absence of CHX, by 9 and 12 h.p.i. there was substantial variation in the number of IBs per cell. In PIV5.F157-infected cells the number of IBs per cell at 9 h.p.i. ranged from 20 to 194, increasing to 38 to 292 IBs per cell by 12 h.p.i. Up until 12 h.p.i. small IBs (≤2.5 µm) made up the vast majority of the total IB population (>98%). However, by 24 h.p.i. the number of small IBs had significantly decreased to an average of 64 IBs per cell whilst the number of large IBs (>2.5 µm) had increased significantly, accounting for approximately 11% of the total IB population. The observation that there is a decrease in the average number of small IBs per cell between 12 and 24 h.p.i. and a concomitant increase in the number of large IBs suggests that large IBs form by fusion of numerous small IBs. Similarly, cells infected with PIV5.S157, which readily establishes persistent infections, display variation at 9 and 12 h.p.i. with the number of IBs ranging from 11 to 195 per cell at 9 h to 85–295 per cell at 12 h.p.i. Comparably to PIV5.F157, at 24 h there is a decrease in the number of IBs to 55 per cell. Simultaneously, the number of larger IBs increased from <2% of the total IB population at 12 h.p.i up to 12% at 24 h.p.i. Time course analysis of persistent PIV5.S157 did not reveal any significant differences in the number or kinetics of IB formation compared to cells infected with lytic/acute PIV5.F157.

**Fig. 2.**
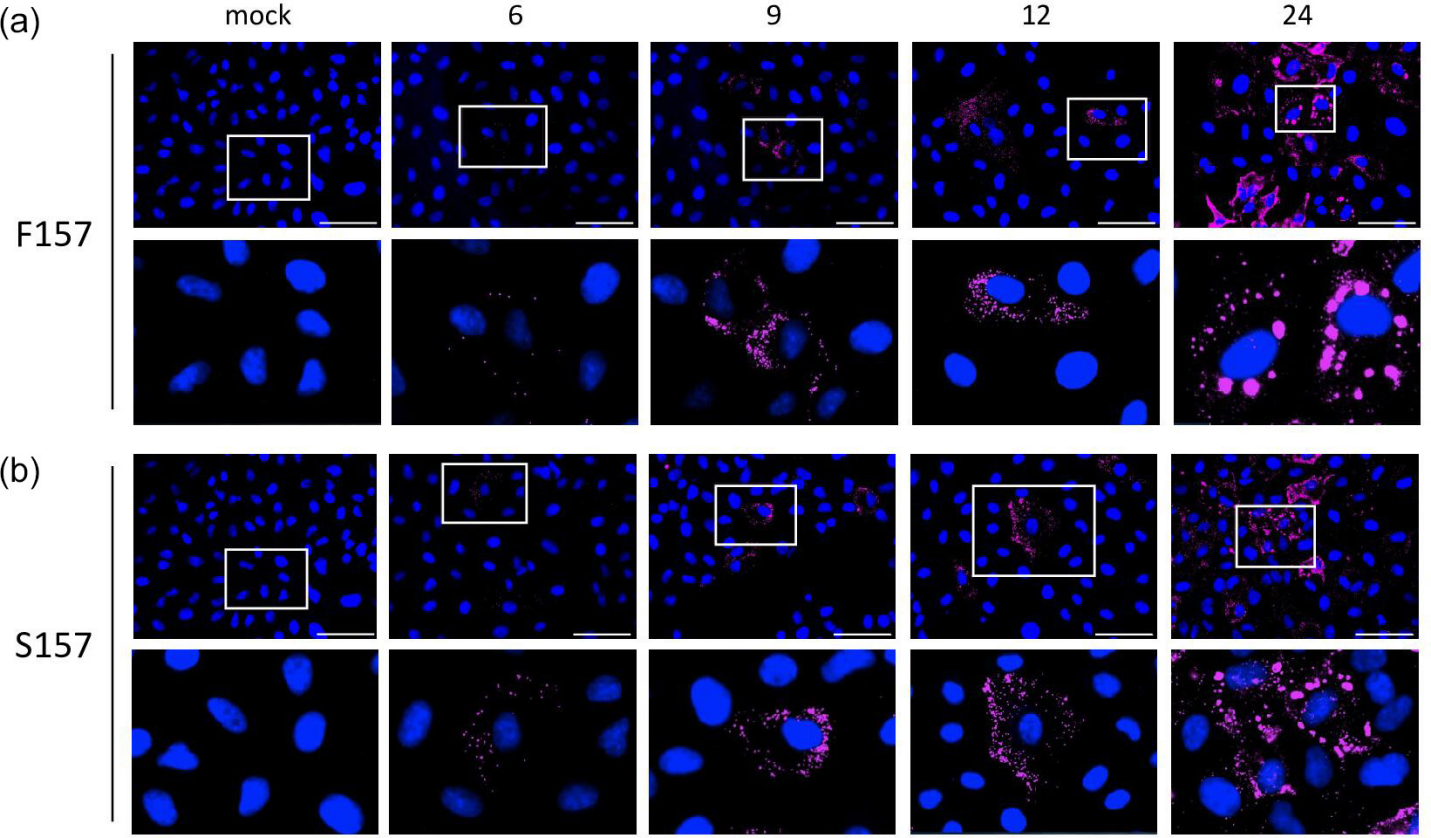
The dynamics of IBs formed during lytic/acute and persistent PIV5 infections over time. (**a, b**) A549 cells seeded on coverslips were infected with PIV5.F157 or PIV5.S157 at an m.o.i. of 0.1 p.f.u. per cell. At the indicated time points post-infection, the cells were fixed, permeabilized and incubated with an anti-NP mAb. The cells were imaged using the Evos M5000. Bar, 75 µm.

To ensure that the observed dynamics of IB formation was not due to a high m.o.i. infection, A549 cells were infected with PIV5.F157 or PIV5.S157 at a low m.o.i. of 0.1 p.f.u. per cell. The cells were fixed at various times post-infection and subsequently immunostained using anti-NP antibody (Fig. 2). Both PIV5.F157 and PIV5.S157 low m.o.i. infections display similar IB formation dynamics. At early times post-infection, 6 h.p.i., a small number of IBs are observed which increase in number up to 12 h.p.i. The IBs observed up to 12 h.p.i. are small. Similarly to high m.o.i. infections, at 24 h.p.i. cells could be observed in which the number of IBs had decreased whilst simultaneously increasing in size. These cells were usually surrounded by cells in which small IBs could be detected, indicating virus spread from the initially infected cells. These results clearly show comparable IB formation dynamics to those observed during high m.o.i. infections.

**Fig. 3.**
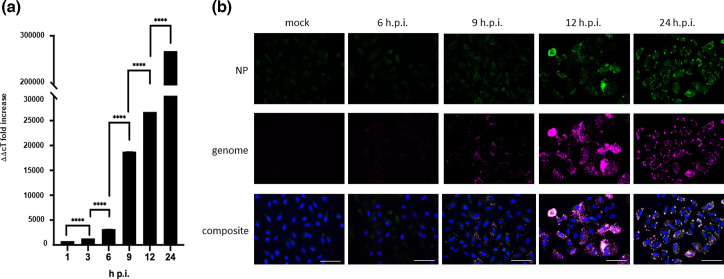
Quantification and localization of virus genomes during PIV5 infection. (**a**) A549 cells grown in 25 cm^2^ flasks were infected with PIV5-F157 at an m.o.i. of 10 p.f.u. per cell. At the indicated time points post-infection, the RNA was extracted and subjected to qRT-PCR to determine the relative abundance of virus genomes. The Ct values were normalized to the housekeeping gene actin. The ΔCt values were compared to uninfected A549 samples to ascertain the ΔΔCt fold change. Statistical analysis was performed using GraphPad (*n*=3 independent experiments). (**b**) A549 cells were grown on chambered slides and infected with PIV5-F157 at an m.o.i. of 10 p.f.u. ml^–1^. At the indicated time points the cells were fixed, permeabilized and incubated with anti-NP antibody for 1 h. The cells were then subjected to RNAscope *in situ* hybridization using a virus genome-specific probe (Cy5). The cells were imaged using the Evos M5000. The anti-NP antibody staining at 6 h.p.i. was below the threshold for detection. Images are representative of *n*=3. Bar, 75 µm.

Our previous studies on the kinetics of PIV5 transcription and replication, using high-throughput sequencing, showed that maximum rates of PIV5 mRNA transcription occurred between 6 and 12 h.p.i., whilst genome replication gradually increased up to 24 h.p.i., although there was clear evidence of genome replication between 6 and 12 h.p.i [10]. Given that there was an increase in IBs as early as between 3 and 6 h.p.i., we next used qRT-PCR to investigate how early genome replication occurred (Fig. 3a). A549 cells were infected with PIV5 at an m.o.i. of 10 p.f.u. per cell and RNA was extracted at various times post-infection. A single positive-sense genome-specific primer for the L gene was used during reverse transcription to ensure only negative-sense viral genomes were transcribed into cDNA and not positive-sense mRNAs/antigenomes. The qRT-PCR revealed a slight, approximately 1.7-fold, but significant increase in the number of viral genomes between 1 and 3 h.p.i. followed by a 2.4-fold increase between 3 and 6 h.p.i. (Fig. 3a). There was a substantial increase of 6-fold in the number of viral genomes between 6 and 9 h.p.i., increasing by a further 1.4-fold by 12 h.p.i. As expected, based on our previous work, the largest increase occurred between 12 and 24 h.p.i. where viral genomes increase by 10-fold (Fig. 3a).

We next used an RNAscope to confirm that viral genomes were present in IBs. A549 cells were infected with PIV5 at an m.o.i. of 10 p.f.u. per cell and fixed at 6, 9, 12 and 24 h.p.i. First, the cells were incubated with anti-NP antibody for 1 h, and thereafter stained using a specific Cy5 fluorescently labelled RNAscope probe to identify viral genomes. The cells were then incubated with an FITC-secondary antibody (green) to visualize the presence of NP within infected cells (Fig. 3b). At 6 h.p.i., the RNAscope demonstrated the presence of viral genomes in small foci throughout the cytoplasm. Unfortunately, although small cytoplasmic foci were observed with standard fluorescence using the anti-NP antibody (Fig. 1), using RNAscope procedures the anti-NP antibody staining was below the threshold of detection up to 9 h.p.i. However, at 12 and 24 h.p.i., anti-NP antibody staining was detected under RNAscope conditions and clearly showed the presence of viral genomes and NP within the cytoplasmic foci (Fig. 3b).

**Fig. 4.**
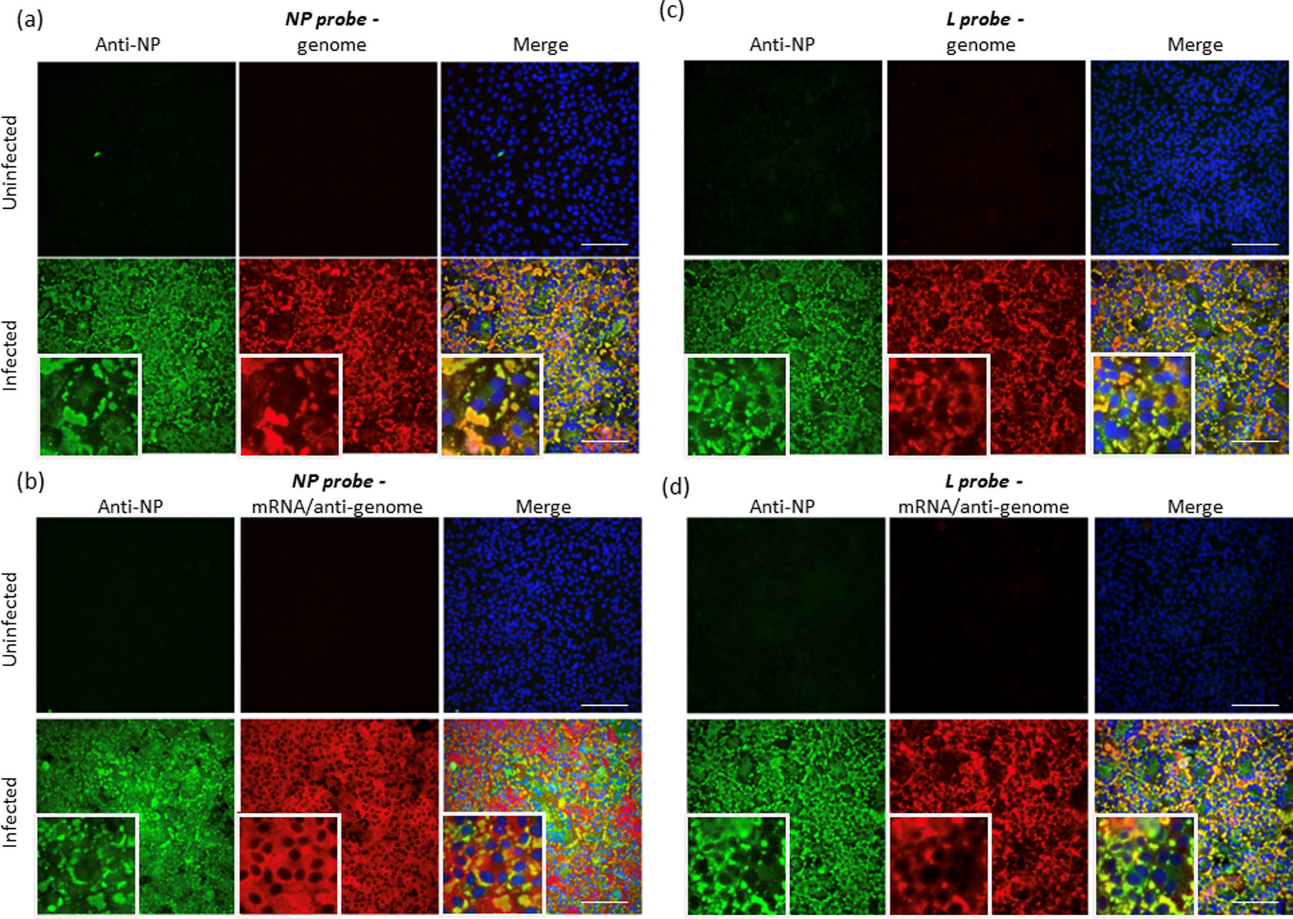
The localization of virus genomes and mRNAs during PIV5 infection. Vero cells were either mock-infected or infected with PIV5 (strain CPI) at a high m.o.i. At 48 h p.i., the cells were fixed and co-stained by immunofluorescence, with an antibody to NP, and by *in situ* hybridization, using probes specific for genomic NP (a) or L (c) RNA or NP (b) or L (d) antigenomic RNA/mRNA. The cells were also counter-stained with DAPI to reveal the location of the nuclei. The merged patterns are from all three stains. Magnified images have been inserted at the bottom left-hand corner of the panels so that the distribution of the stain within individual cells can be visualized. Cells were visualized using a Leica DM5000B wide-field fluorescence microscope using a ×20 objective. Note: NP and L probes that bind to the genome give the same intensity of staining, whilst the NP probe that binds to mRNA/antigenomes give more intense staining than the L probe that binds to mRNA/antigenomes as the abundance of the NP mRNA is significantly greater than that of the L mRNA.

To ensure that similar dynamics of IB formation were observed with other strains of PIV5, similar experiments were undertaken with the CPI strain. These results supported, and were confirmed by, *in situ* hybridization data [34] (Figs4  5) using probes specific for negative-sense genomic NP and L sequences. The majority of the genome staining is confined to IBs. However, labelling was not restricted solely to IBs in that a diffuse cytoplasmic signal was detected throughout the cytoplasm [34] (Figs4  5). *In situ* hybridization, using NP and L probes specific for positive-sense mRNA and antigenomes, strongly suggested that IBs also contain antigenomes, but that mRNA is primarily distributed throughout the cytoplasm. This was inferred from the observation that diffuse cytoplasmic staining was primarily observed using the genomic NP-specific probe. In contrast, there was significantly less diffuse cytoplasmic staining with the antigenomic L-specific probe, but IBs could be readily detected (Fig. 4). This would be predicted from the fact that there is significantly more NP mRNA present in infected cells than L mRNA, but that the NP and L probes should be equally as effective in detecting antigenomes [10].

**Fig. 5.**
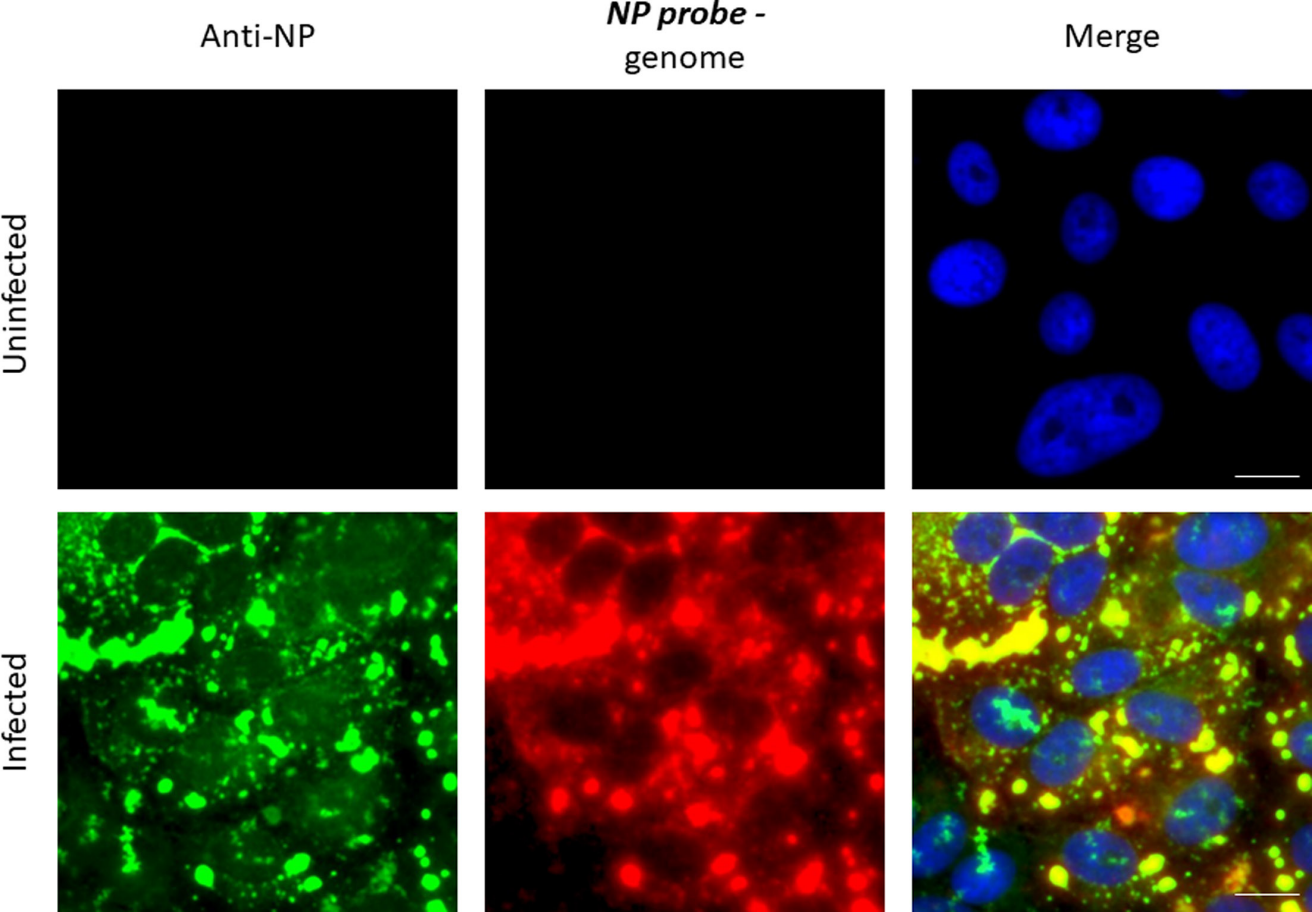
Virus genomes are located in cytoplasmic IBs and are diffusely distributed throughout the cytoplasm. Vero cells were either mock-infected or infected with PIV5 (strain CPI) at a high m.o.i. At 48 h p.i., the cells were fixed and co-stained by immunofluorescence, with an antibody to NP, and by *in situ* hybridization, using probes specific for genomic NP. The cells were also counter-stained with DAPI to reveal the location of the nuclei. The merged patterns are from all three stains. Cells were visualized using a Leica DM5000B wide-field fluorescence microscope using a ×40 objective. Note: *in situ* hybridization shows both diffuse cytoplasmic staining and staining of IBs indicating that viral genomes are present within IBs and more diffusely distributed throughout the cytoplasm of infected cells. Bar, 25 µm.

### Newly synthesized RNA can be detected in some, but not all, PIV5 IBs

**Fig. 6.**
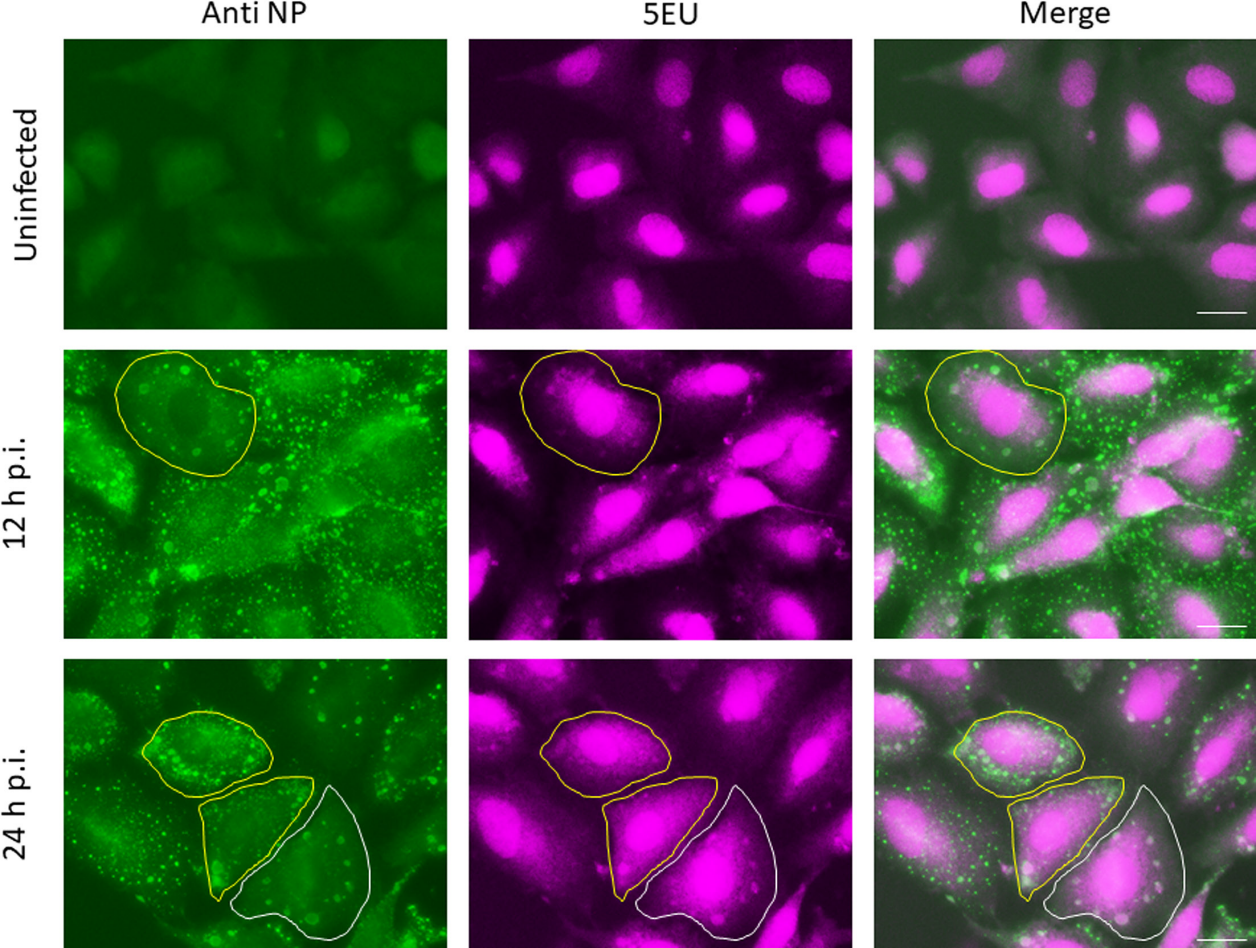
The synthesis of viral RNA during PIV5 infection. A549 cells were either mock infected or infected with PIV5. At 12 and 24 h.p.i., cells were incubated with 5EU for 1 h and then fixed. The cells were then incubated with anti-NP mAb (green). The 5EU was detected using Alexa Fluor 647-azide (cyan). The cells were imaged using the Evos M5000. Bar, 25 µm.

To visualize the location of newly synthesized RNA, A549 cells were infected with PIV5.F157 and subjected to nascent RNA labelling using 5EU incorporation at 12 and 24 h which was subsequently detected by AlexaFluor 647-labelled dye (Fig. 6). The cells were also counter-stained with an antibody to NP to visualize the presence of IBs. Unfortunately, we could not perform these experiments in the presence of actinomycin D, to inhibit host cell transcription, as actinomycin D also inhibited PIV5 replication. As a consequence, and as expected, strong nuclear staining was observed in all cells, reflecting ongoing host cell transcription. However, very little cytoplasmic staining was observed in uninfected cells. In contrast, significantly more diffuse cytoplasmic staining was observed in PIV5-infected cells that presumably reflects the presence of newly synthesis viral mRNA diffusely distributed throughout the cytoplasm (as observed in the *in situ* hybridization studies). Although the RNAscope and *in situ* hybridization data showed that all IBs contained viral genomes, 5EU staining was observed in some, but not the majority of, IBs (Fig. 5). The presence of 5EU staining in some IBs presumably reflects either the ongoing replication of genomes or the accumulation of newly synthesized genomes into IBs. In either situation, the fact that not all IBs stain with 5EU strongly suggests that in 5EU-negative IBs there was little or no ongoing virus transcription or replication.

### Disruption and reformation of PIV5 IBs upon osmotic shock

**Fig. 7.**
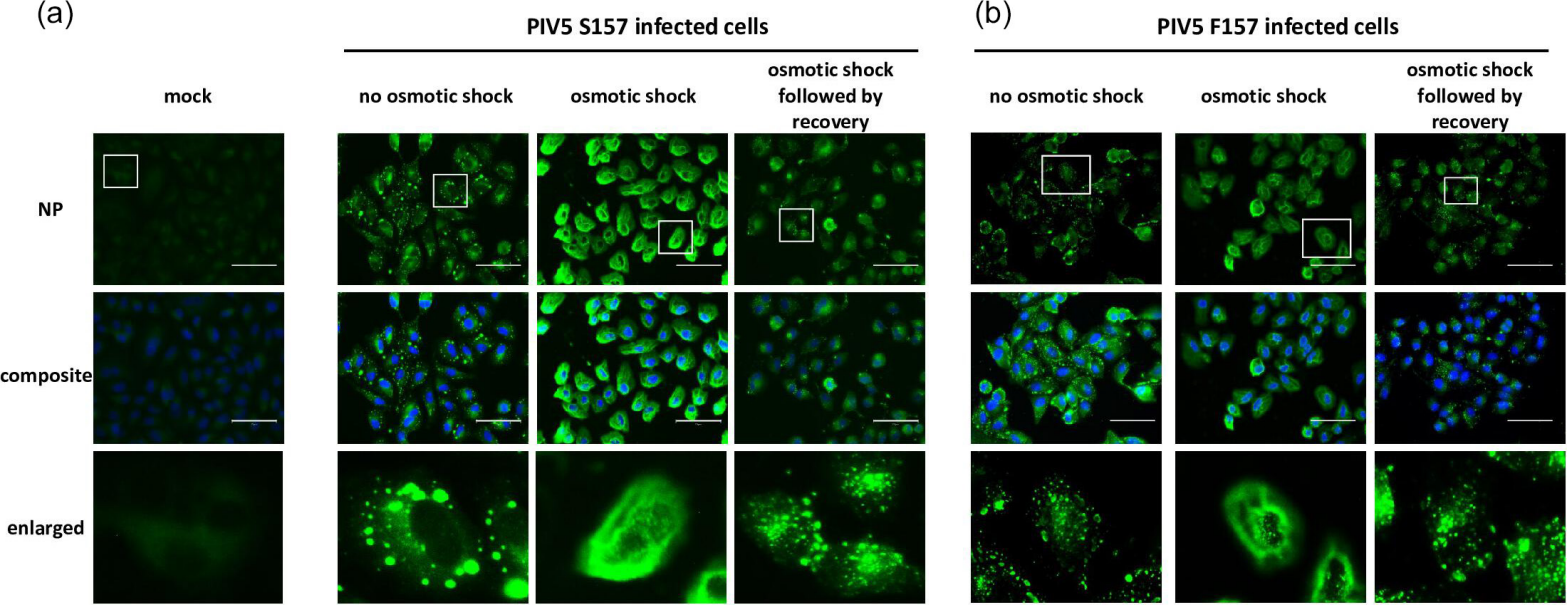
The IBs generated during lytic/acute and persistent PIV5 infections are dynamic liquid–liquid organelles. A549 cells grown on coverslips were infected with PIV5.F157 (lytic/acute) or PIV5.S157 (persistent) at an m.o.i. of 10 p.f.u. per cell. At 24 h.p.i., the cells were subjected to osmotic shock by incubating the cells in a dilution of DMEM and water at a ratio of 1 : 5 respectively for 10 min. Where specified the cells were then allowed to recover by incubating the cells with DMEM for 10 min. The cells were fixed, permeabilized and incubated with an anti-NP mAb. The nucleus was stained with DAPI. The cells were imaged using the Evos M5000. Images are representative of *n*=3. Bar, 75 µm.

We have previously shown that PIV5 IBs can be disrupted by cold shock [34], whilst others have used osmotic shock to disrupt Mononegavirales IBs, demonstrating that they have the property of liquid organelles. To confirm that PIV5 IBs can also be disrupted by osmotic shock, as well as cold shock, A549 cells that had been infected with PIV5.S157 or PIV5.F157 for 24 h were incubated with hypotonic shock media for 10 min only or were allowed to recover by incubation in DMEM at 37 °C for 10 min. The cells were then fixed and analysed by immunofluorescence using an anti-NP antibody and compared to untreated infected cells (Fig. 7). As expected, the osmotic shock readily disrupted the IBs present in the PIV5-infected cells, confirming their characteristics as membrane-less liquid organelles. Recovery of the cells in DMEM for as little as 10 min after osmotic shock resulted in the re-formation of some IBs, further highlighting their fluid and dynamic nature. Since very little virus transcription and replication would have occurred within the 10 min recovery time, these observations suggest that formation of IBs is not dependent upon ongoing virus transcription and replication, but rather on the self-assembly properties of the viral replication proteins and nucleocapsids.

### Disruption of IBs in persistently infected cells does not reactivate viral RNA synthesis

**Fig. 8.**
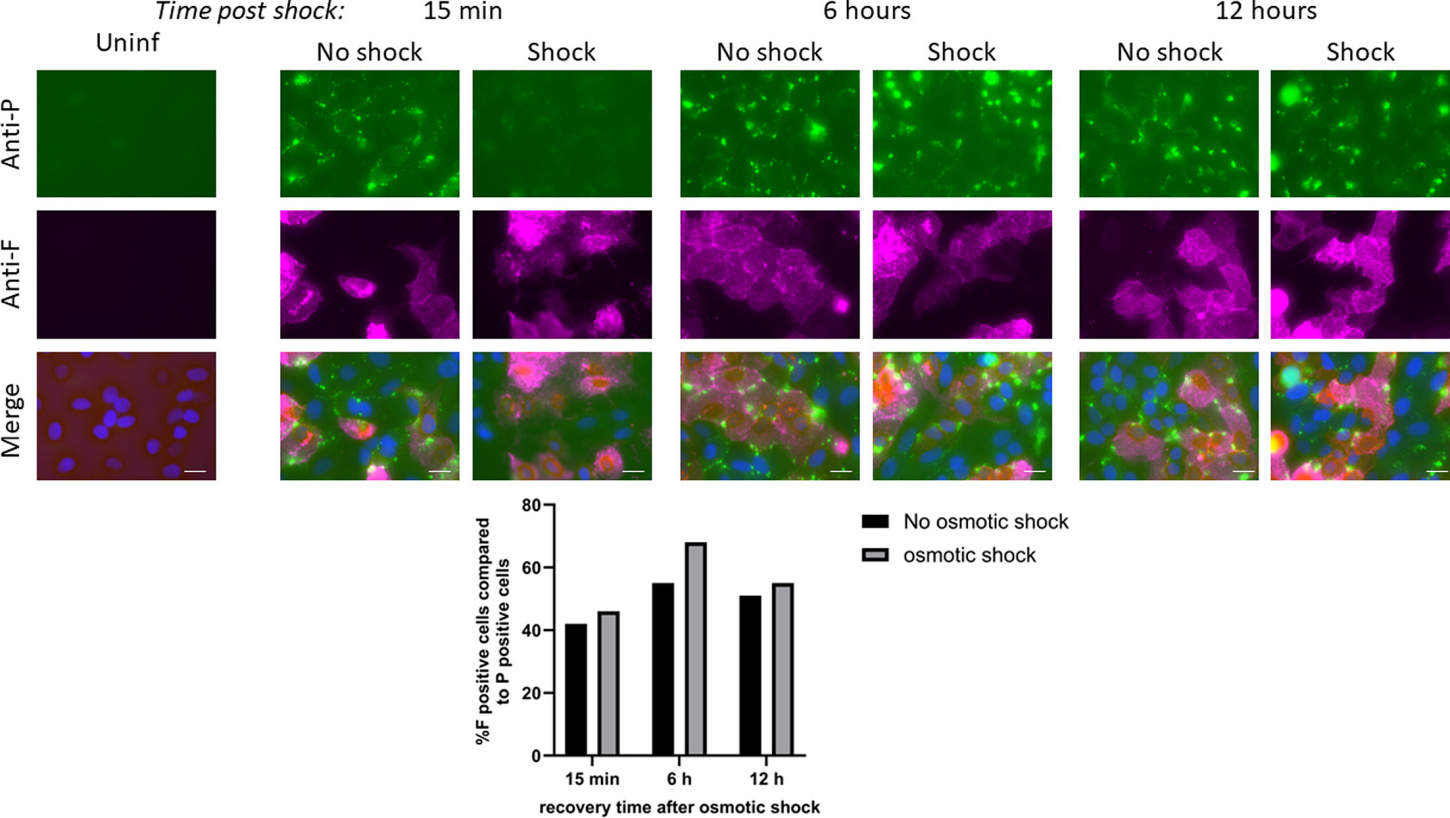
Disruption of PIV5.S157 IBs does not reactivate viral RNA synthesis. To establish a persistent infection A549 cells were infected with PIV5.S157 at an m.o.i. of 10 p.f.u. per cell, and after 4 days the cells were seeded onto coverslips and subjected to osmotic shock for 10 min as previously described. The cells were then incubated with DMEM and allowed to recover for the time indicated post-osmotic shock. The coverslips were fixed, permeabilized and subsequently incubated with an anti-F antibody (Cy5) and anti-P antibody (GFP). The nucleus was stained with DAPI. The cells were imaged using the Evos M5000. Images are representative of *n*=3. Bar, 25 µm. B) The percemtage of F positive cells compared to P positive cells was quantified using ImageJ.

We have previously shown that PIV5 IBs can readily be detected in persistently infected cells at times when there is very little ongoing transcription or replication within the majority of cells. We therefore next investigated whether osmotically shocking persistently infected cells to ‘release’ virus genomes from IBs would reactivate virus transcription and replication. It is possible to distinguish between cells in which active transcription is occurring, or has recently occurred, from cells in which it is repressed by staining persistently infected cells with antibodies to the HN or F proteins. This is because the HN and F proteins are rapidly turned over and so although all persistently infected cells contain IBs that can be stained with an anti-NP antibody, many cells will be negative for F or HN [42]. Persistently infected A549 cells were either osmotically shocked for 15 min or mock-treated, and subsequently allowed to recover by incubation with DMEM at 37 °C for 0, 6 or 12 h, and fixed and stained for NP and F (Fig. 8). As predicted, osmotic shock disrupted the IBs, which then re-formed with time. However, there was no evidence that osmotically shocking the cells led to a reactivation of the virus since a similar ratio of cells that were positive for NP and negative for F was present in cells that had or had not been osmotically shocked. These results suggest that most of the virus genomes in IBs present in persistently infected cells are in a repressed state that cannot be reactivated by disruption of the IBs

## Discussion

The data presented here on PIV5 IBs in general support the observations by others on the properties of Mononegaviralae IBs, whilst raising a number of questions as to their nature and importance in particular with regard to persistent infections. Similar to that which was originally shown in RABV infection, and subsequently shown for other Mononegaviralae including paramyxoviruses MeV and PIV3 [21,34], small numbers of small PIV5 IBs are formed during the initial stages of infection with their numbers increasing until approximately 12 h.p.i., after which time the small IBs begin to merge to form larger IBs. Consistent with the model which requires that there must be some genome replication for an increase in IB numbers, using qPCR we show there is a small increase in genome numbers as early as 3–6 h.p.i. Virus replication then increases reaching maximum rates, as previously observed by high-throughput sequencing, between 12 and 24 h.p.i [10]. These results demonstrate that virus replication must begin at very early times post-infection and at times before NP has accumulated to large amounts throughout the cell. Since the switch between transcription and replication is dependent upon the concentration of NP, these results are consistent with the model, first proposed for RABV, in which during initial stages of infection, transcription and viral protein synthesis occur at restricted sites around incoming genomes [21]. The local concentration of NP is then thought to be sufficient to facilitate genome replication and as a consequence, with time, these sites develop into small IBs that have the property of liquid organelles, where virus transcription and replication continues. Subsequently, the model proposes that individual nucleocapsids are released from the initial IBs and are transported to new sites within the cytoplasm to form new sites of transcription and replication that result in the formation of new IBs. As the number and size of these IBs increase, they presumably begin to fuse forming significantly larger IBs. This latter phase correlates with maximum rates of virus replication. Work on RSV suggests that viral mRNA synthesis also occurs within IBs [22]. However, our *in situ* hybridization data on PIV5-infected cells show that viral mRNA is distributed throughout the cytoplasm. It therefore seems unlikely that significant amounts, if any, of PIV5 mRNA accumulates within PIV5 IBs, although presumably at early times post-infection there are sufficient levels of transcription and NP synthesis around the incoming genomes to facilitate early replication. Rather, the increase in the relative amount of NP localized within the IBs may be a consequence of its encapsidation during replication around newly synthesized virus genomes. However, it is also important to note that the formation of IBs is not dependent upon ongoing virus transcription and replication, as after osmotically shocking the cells, IBs begin to reform rapidly and much faster than would be the case if newly synthesized virus protein synthesis was required. Thus, the formation of IBs may not necessarily be a direct consequence of virus replication but may be a consequence of the tendency of nucleocapsids, and NP in the absence of soluble V, and P to associate resulting in the formation of IBs. The *in situ* hybridization data also show that virus genomes can be detected outside IBs with some being diffusely distribution throughout the cytoplasm. Whether these genomes are being exported from IBs for packaging and export from the cell as virions or whether they are actively involved in virus transcription or replication is currently unclear.

It is also unclear what the nature and role of PIV5 IBs are in the establishment and maintenance of persistence. It is clear that most IBs must be relatively inactive in persistently infected cells, as highlighted following infection with PIV5.S157; at 96 h.p.i., the levels of viral transcription and replication had reduced to minimal levels [42. Yet, IBs are readily detected in these cells despite the absence of HN and F proteins, demonstrating that IBs are not always active areas of virus replication. More surprisingly, it appears that many IBs may also become inactive during the early phases of virus infection, as even at 12 and 24 h.p.i. many IBs did not incorporate detectable levels of 5EU.

IBs in persistently infected cells retain the property of liquid organelles as they can still be disrupted by osmotic shock. Since we had previously speculated that IBs may be reservoirs in which the virus can remain hidden from innate intracellular anti-viral responses, but from which it may become reactivated (hence the observation that the virus fluxes between active and repressed states in individual cells), we speculated that osmotically shocking persistently infected cells may release virus genomes from the IBs resulting in an increase in virus transcription and replication. However, there was no obvious increase in the number of cells positive for expression of F in persistently infected cells following osmotic shock (Fig. 7). It thus appears that in persistently infected cells the majority of virus genomes in the IBs must be in an inactive form, presumably due to the phosphorylation of P at critical residues, and cannot be activated simply by releasing them from the IBs [42]. In this regard, it is of note that the persistent genome phenotype is dominant over the lytic/acute phenotype [44]. It may therefore be that whilst some of the genomes in IBs have the potential to become active if released individually by chance from an IB, the release of all genomes from the IBs through osmotic shock, including those with a persistent phenotype, and the rapid reformation of IBs after osmotic shock may prevent reactivation of virus replication. It is thus still unclear as to how PIV5 is reactivated in persistently infected cells, what the role of IBs is in the maintenance of persistence, and what are the virus, host cell and environmental factors that influence virus reactivation.

## Abbreviations

CPE: cytopathic effect
DMEM: Dulbecco's modified Eagle medium
EBOV: Ebola virus
5EU: 5-ethynyl-uridine
HMPV: human metapneumovirus
h.p.i.: hours post-infection
IB: inclusion body
L: large
Le: leader
MeV: measles virus
MuV: mumps virus
NiV: Nipah virus
NP: nucleoprotein
P: phosphoprotein
pen/strep: penicillin and streptomycin
PIV3: parainfluenza virus 3
PIV5: parainfluenza virus 5
qRT-PCR: quantitative reverse transcriptase PCR
RABV: rabies virus
RdRp: RNA-dependent RNA polymerase
RSV: respiratory syncytial virus
Tr: trailer
VSV: vesicular stomatitis virus
